# Response of Soil Microbial Community to Vegetation Reconstruction Modes in Mining Areas of the Loess Plateau, China

**DOI:** 10.3389/fmicb.2021.714967

**Published:** 2021-08-25

**Authors:** Jiao Zhao, Jing Ma, Yongjun Yang, Haochen Yu, Shaoliang Zhang, Fu Chen

**Affiliations:** ^1^Engineering Research Center of Ministry of Education for Mine Ecological Restoration, Xuzhou, China; ^2^Low Carbon Energy Institute, China University of Mining and Technology, Xuzhou, China

**Keywords:** vegetation reconstruction, soil bacterial, soil fungal, ecological restoration, damaged mine

## Abstract

Vegetation reconstruction and restoration is vital to the health of the mine land ecosystem. Different vegetations might change microbial community structure and function of soil, mediating the biogeochemical cycle and nutrition supply to the soil. To clarify the response of soil microbes to different vegetation reconstruction modes in the mining areas of the Loess Plateau, China, soil microbial community structures and functions were determined by the MiSeq high-throughput sequencing along with PICRUSt2 and FUNGuild tools. The fungal community richness was observed to be the highest in grassland soil and positively correlated with soil organic matter, total nitrogen, and nitrate-nitrogen. The bacterial and fungal community structures were similar in grassland and brushland areas, but were significantly differentiated in the coniferous and broadleaf forest, and the leading factors were soil pH and nitrate-nitrogen. Actinobacteriota, Proteobacteria, and Acidobacteriota were the dominant bacterial phyla under different vegetation reconstruction modes. The dominant phyla of fungi were Ascomycota, Basidiomycota, and Mortierellomycota. Different vegetation reconstruction modes did not affect the bacterial functional communities but shaped different functional groups of fungi. The grassland soil was dominated by saprotrophic fungi, while symbiotrophic fungi dominated the coniferous and broadleaf forests. The results suggested that shifts in vegetation reconstruction modes may alter the mining soil bacterial and fungal community structures and function. These findings improve the understanding of microbial ecology in the reclaimed mine soil and provide a reference for the ecological restoration of fragile mining ecosystems.

## Introduction

Mining activities usually adversely disturb the land surface, thus changing the soil ecological environment (Wang et al., 2014) and posing an unfavorable influence on soil microbial community (Li J. G. et al., 2014), especially in the arid and semiarid areas, which are ecologically vulnerable. The Loess Plateau of China, as one of the areas with the most fragile environment in the world, is well-known for its severe soil erosion and vegetation degradation (Zhang et al., 2011). Twenty years ago, it was the most important coal production base in the world but is disturbed greatly by mining. Vegetation reconstruction has been an important marker of ecological restoration of the damaged mine. The ultimate goal of mine land reclamation is to restore the productivity of the land postmining and maintain sustainable development of the ecosystem (Zhang et al., 2011; Li J. G. et al., 2014). Restoration of soil microbial communities plays a vital role in driving a variety of ecosystem functions and ecological processes (Cheng et al., 2020) and is thus a key step toward achieving sustainable soil restoration in mining areas (Dangi et al., 2012).

Soil microbes play a vital role in the recycling of soil nutrients and influence soil organic matter turnover, nutrient transformations, and biogeochemical cycling (Falkowski et al., 2008; Yang and Zhu, 2015; Baldrian, 2017), as well as plant productivity (Chen et al., 2020). For example, free-living nitrogen-fixing bacteria (e.g., *Pseudomonas*, *Azospirillum*, and *Azotobacter*) inhabiting soils can fix a large amount of nitrogen (Burgmann et al., 2004). Rhizobacteria were generally thought to have a positive influence on plant performance *via* their ability to enhance phosphorus mobilization, carry out atmospheric nitrogen fixation, and sequester iron by producing siderophores and phytohormones (Abou-Shanab et al., 2019). Members of the genera *Rhizobium*, *Bacillus*, *Microbacterium*, *Arthrobacter*, and *Pseudomonas* were previously reported as a metal-resistant rhizobacteria and/or endophytes (Abou-Shanab et al., 2010). Arbuscular mycorrhizal fungi improve the supply of soil nutrients to plants and increase the tolerance of plants to nutrient-poor soil (Johnson et al., 2010). Soil microbial diversity is helpful in maintaining the function of the ecosystem (Srivastava et al., 2012; Delgado-Baquerizo et al., 2016). However, microbial diversity changes with different vegetation restoration categories as well as land use types (Thomson et al., 2015; Zhang et al., 2016; Xiong et al., 2021).

In some earlier studies, the whole structure of soil microbial community was found to follow the change of aboveground plant community (Peay et al., 2013; Barberan et al., 2015). However, some studies have shown that vegetation category does not influence the underground community (Lekberg and Waller, 2016; Tedersoo et al., 2016). The influence of the specific vegetation type on soil microbial community was found to be closely related to soil category (Bulgarelli et al., 2012) and was sometimes stronger than the specific function of soil, which could explain to a large extent, the variation in the fungal community composition (Urbanová et al., 2015). The relationship between soil and vegetation in the mine ecological restoration process is very complex, which is not only limited by local natural environmental factors but also influenced by an alien vegetation and has not been clearly examined until now. The illustration of the response of structure and function of soil microbial community to vegetation restoration is helpful in understanding the resilience and the self-sustainability mechanism of the damaged mine ecosystem.

Different vegetation categories influence the quality and the quantity of soil organic matters in terms of litter decomposition and root secreta (Bais et al., 2006). Moreover, different carbon pools of soil affect the richness and composition of soil microbial communities to varying degrees (Augusto et al., 2015). Nevertheless, vegetation restoration has been widely used in mining areas, but the response of soil microbial community structures and their functions to different vegetations has not yet been well characterized in the mining areas of the Loess Plateau, China. Herein, the objectives of this study were to (1) evaluate the variations in bacterial and fungal community structures and functions under different vegetation reconstruction modes and (2) examine the dominant environmental factors and their influence on microbial community structures and functions. The aims of this study were to enhance our understanding of soil microbial community functions and adaptability in mining areas and to provide a scientific basis for ecological restoration in the mining areas of the Loess Plateau and the construction of green mines.

## Materials and Methods

### Study Area and Soil Sampling

The study area is located at the Heidaigou opencast coal mine, in Jungar Banner, Inner Mongolia, which is in the northern margin of the Loess Plateau (39°43′N–39°49′N, 111°13′E–111°20′E). It has a typical temperate continental climate with an annual temperature of 7.2°C and an annual precipitation of 391.6 mm. The rain mainly falls between July and September, accounting for 70% of the precipitation of the entire year. The annual evaporation is 1,824.7–2,896.1 mm. The altitude is 1,025–1,302 m, having typical hilly and gully area of the Loess Plateau. The total land area under study was 5,124 hm^2^. There were five waste disposal sites in the opencast mine of Heidaigou, which have been in continuous reclamation since 1995 (Lei et al., 2016). The eastern garbage dump, which has been reclaimed since 1998, was chosen as our study target with an area of 210 hm^2^. Reclamation with three layers with a layer gap of 30 m was conducted. The altitude of the lowest layer was 1,215 m.

**FIGURE 1:**
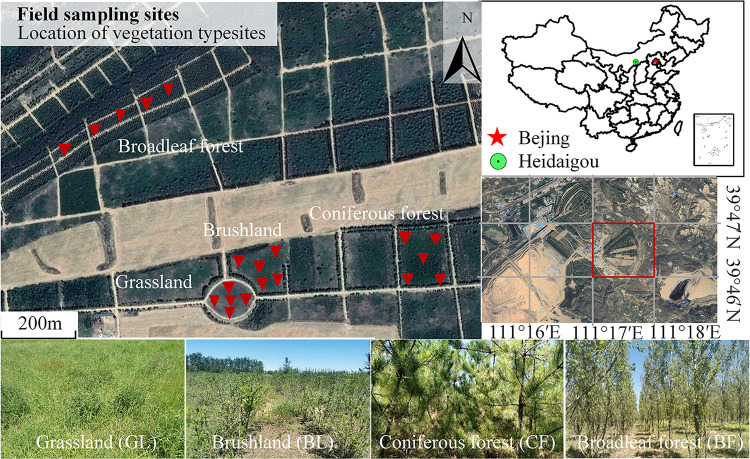
The landscape of vegetation rehabilitation in the east dump of Heidaigou opencast coal mine. The red triangle indicates the location of the sampling point, and the red square is the east dumping site of Heidaigou in the study area.

We investigated the eastern garbage dump and collected soil samples from four main vegetation reconstruction modes including grassland (GL), brushland (BL), coniferous forest (CF), and broadleaf forest (BF) (Figure 1). The grassland is dominated by *Medicago sativa* and *Stipa bungeana*. The bushes are mainly composed of *Hippophae rhamnoides*. The coniferous forest consists of *Pinus tabulaeformis*, and the broadleaf forest consists of *Populus simonii*. Soil samples were collected between July 28 and August 4 in 2020. The investigated area of GL was a square of 1 m × 1 m. However, the corresponding areas of BL, CF, and BF were 10 m × 10 m each. Before sample collection, the litter from the topsoil was removed. Nearly 200 g topsoil was collected randomly from a depth of 0 to 10 cm from the surface of five cores of each plot. Thus, the mixture contained nearly 1,000 g of sample. All 20 samples were then immediately transported on ice to the laboratory. Stones, as well as plant and animal residues, were removed from soil samples using a 2-mm mesh. The soil sample was then divided into three parts. One fresh portion was used to isolate DNA for biodiversity analysis. The second portion was kept at less than 4°C for the analysis of soil enzyme activity and physicochemical properties. The third portion was air-dried indoor for the analysis of soil chemical properties.

### Analyses of Soil Physicochemical Properties and Enzyme Activities

Soil temperature was measured on-site using a temperature and humidity meter by a rapid measurement method (Guangdong Shunkeda, TR-6). Soil pH was measured using a glass electrode in a 1:2.5 (w/v) soil:water suspension (Rayment and Higginson, 1992). Soil organic matter (SOM) was determined by a colorimetric method, using hydration heat during the oxidation of potassium dichromate (Barahona and Iriarte, 2001). Soil total nitrogen (TN) was determined by Kjeldahl’s digestion method (Tsiknia et al., 2014). Soil nitrate-nitrogen (NO_3_^–^-N) was determined by calcium chloride extraction-ultraviolet spectrometry (Barahona and Iriarte, 2001). Available phosphorous in soil was extracted by sodium bicarbonate and determined using the molybdenum blue method (Olsen, 1954). Soil enzyme activities were spectrophotometrically determined (Tabatabai, 1994) and described by Yin et al. (2014). The β-glucosidase activity was measured using *p*-nitrophenyl-β-D-glucopyranoside (PNPG) as a substrate, and after incubation at 37°C for 1 h, the amount of *p*-nitrophenol (PNP) was measured at 410 nm. Soil urease was determined by the sodium hypochlorite-phenol colorimetric method using urea as the substrate, and after incubation at 37°C for 24 h, the amount of NH_4_^+^ was measured at 578 nm. Soil leucine aminopeptidase activity (S-LAP) was measured using a commercially available kit (Solarbio Science & Technology Co., Ltd., Beijing, China). Soil alkaline phosphatase activity was measured using disodium phenyl phosphate as a substrate, and after incubation at 37°C for 24 h, the amount of phenol was measured at 570 nm.

### Soil DNA Extraction, PCR Amplification, and MiSeq Sequencing

Soil total DNA was extracted from 0.25 g fresh soil using the E.Z.N.A.^®^ soil DNA Kit (Omega Bio-tek, Norcross, GA, United States) according to the protocol of the manufacturer. The final DNA concentration and purification were determined by NanoDrop 2000 UV–vis spectrophotometer (Thermo Scientific, Wilmington, DE, United States), and DNA quality was checked by 1% agarose gel electrophoresis.

The hypervariable region V3–V4 of the bacterial 16S rRNA gene and the ITS1 region of the fungal rRNA gene were amplified from the isolated DNA using primer pairs 338F (5′-ACTCCTACGGGAGGCAGCAG-3′) and 806R (5′-GGACTACHVGGGTWTCTAAT-3′) (Xu et al., 2016) and with primer pairs ITS1F (5′-CTTGGTCATTTAGAGGAAGTAA-3′) and ITS2R (5′-GCTGCGTTCTTCATCGATGC-3′) (Adams et al., 2013), respectively, on an ABI GeneAmp^®^ 9700 PCR thermocycler (ABI, CA, United States). PCR reaction system was prepared as follows: 4 μl of 5 × FastPfu buffer, 2 μl of 2.5 mM dNTPs, 0.8 μl of each primer (5 μM), 0.4 μl TransStart^®^ FastPfu DNA polymerase, and 10 ng DNA template, supplemented with double steamed water to make the total volume of 20 μl. The conditions of the PCR reaction were as follows: (1) 95°C 3 min; (2) a: 95°C 30 s, b: 55°C 30 s, and c: 72°C 45 s, in a total of 27 cycles; and (3) 72°C 10 min. The resulting PCR products were extracted from a 2% agarose gel and purified using the AxyPrep DNA Gel Extraction Kit (Axygen Biosciences, Union City, CA, United States). The obtained purified PCR products were quantified using QuantiFluor^TM^-ST (Promega, United States) according to the protocol of the manufacturer. Purified amplicons were pooled in equimolar ratio and paired-end sequenced (2 × 300) on an Illumina MiSeq platform (Illumina, San Diego, CA, United States) according to the standard protocols by Majorbio Bio-Pharm Technology Co., Ltd. (Shanghai, China).

The raw 16S rRNA and ITS rRNA gene sequencing reads were demultiplexed, quality-filtered by Trimmomatic, and merged by Fast Length Adjustment of SHort reads (FLASH). The reads were truncated at any site receiving an average quality score <20 over a 50-bp sliding window. Primers were exactly matched allowing two nucleotide mismatching, and reads containing ambiguous bases were removed. Based on the default parameters, the Deficiency of Adenosine Deaminase 2 (DADA2) or Deblur in the QIIME2 process was used to reduce the noise of the optimized sequence after quality control splicing (Bolyen et al., 2019). The sequence after DADA2 (or Deblur) noise reduction processing is usually called the amplicon sequence variant (ASV). Follow-up data analysis was performed through the diversity cloud analysis platform (QIIME2 process) of Majorbio Bio-Pharm Technology Co., Ltd. (Shanghai, China).

### Statistical Analysis and Processing

A one-way analysis of variation (ANOVA) and a least significant difference (LSD) multiple comparisons (*P* < 0.05) were used to assess the significant effects of vegetation reclamation types on soil physicochemical properties, enzyme activities, and microbial alpha diversity in R. Principal coordinates analysis (PCoA) based on Bray–Curtis distances was used to evaluate the overall differences in the microbial community structures. The significant difference between bacterial and fungal groups at the genus level was evaluated by the Kruskal–Wallis *H* test. In addition, the relationship between microbial community and environmental factors was analyzed by the redundancy analysis (RDA) and the canonical correspondence analysis (CCA). Functional diversity of bacteria and fungi was predicted using PICRUSt2 (Douglas et al., 2020) and FUNGuild (Nguyen et al., 2016) tools, respectively. Alpha diversity, PCoA, and RDA/CCA of microbes and differences in the functional prediction of microbial species were conducted on the Majorbio Cloud Platform^1^ available freely online.

## Results

### Soil Physicochemical Properties and Enzyme Activities

The soil physicochemical properties showed significant (*P* < 0.05) differences across vegetation reconstruction modes (Supplementary Table 1). Under these modes, CF exhibited the lowest soil temperature. The pH of GL and BL soils was significantly lower than that of CF and BF, but the SOM content, TN, and NO_3_^–^-N were higher than those of CF and BF. Available phosphorous content was the highest in BL soil and the lowest in GL soil.

**FIGURE 2:**
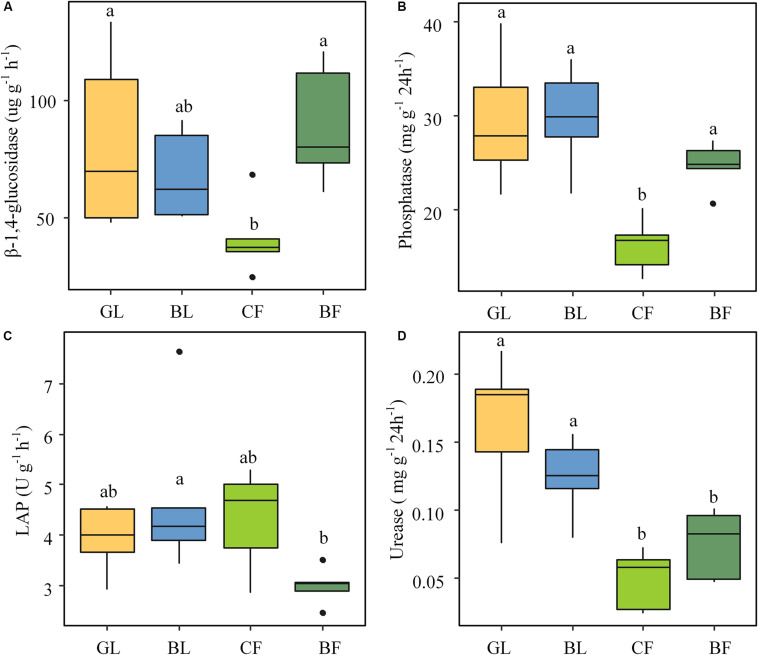
Soil enzyme activities under different vegetation rehabilitation modes: β-glucosidase **(A)**, phosphatase **(B)**, leucine aminopeptidase referred to as LAP **(C)**, and urease **(D)**. The lowercase letters indicate significant differences (*P* < 0.05) among all vegetation rehabilitation modes, based on one-way ANOVA followed by the LSD test. GL, grassland; BL, brushland; CF, coniferous forest; BF, broadleaf forest.

The soil enzyme activities differed significantly (*P* < 0.05) across different vegetation reconstruction modes. Activities of β-glucosidase and phosphatase were significantly lower in CF than those in GL and BF soils (Figures 2A,B). The leucine aminopeptidase (LAP) activity was the lowest in BF among different vegetation reconstruction modes (Figure 2C). The urease activity of GL and BL was significantly higher than that of CF and BF (*P* < 0.05); particularly, the urease activity of GL was 69.5% higher than that of CF (Figure 2D). Except for LAP, soil enzyme activities involved in carbon, nitrogen, and phosphorus cycles of soil under the vegetation reconstruction modes of GL and BL as well as BF were dramatically higher than those of CF. Furthermore, RDA results indicate that activities of urease and phosphatase of GL soil were significantly positively correlated with SOM, TN, and NO_3_^–^-N (Supplementary Figure 1).

### Comparison Between Bacterial and Fungal Diversity and Community Compositions Among Vegetation Reconstruction Modes

**FIGURE 3:**
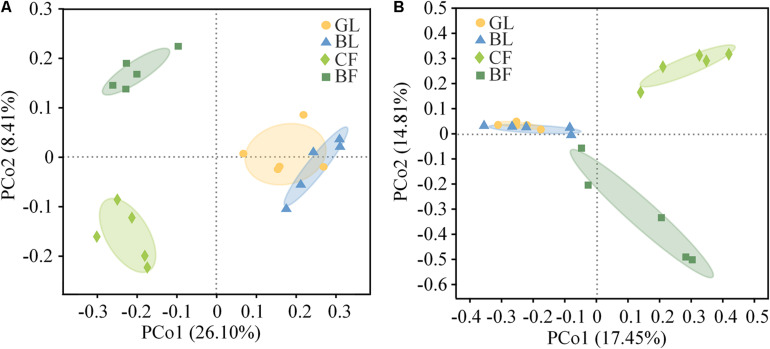
Principal coordinates analysis (PCoA) based on the Bray–Curtis distance of the bacterial communities **(A)** and fungal communities **(B)** in soil.

The difference among Shannon indexes and the observed richness (Sobs) of soil bacterial and fungal community in different vegetation was not dramatic (Supplementary Table 2). However, the Sobs of the fungal community in GL, BL, BF, and CF demonstrated a gradually decreasing trend. The fungal community Sobs in GL was 50.7% higher than that of CF. Although the difference among Shannon indexes of fungal community in different vegetation reconstruction modes was not dramatic, a similar decreasing trend was observed compared with that of fungal community Sobs (Supplementary Table 2). PCoA based on Bray–Curtis distance clearly showed the changes of bacterial and fungal community structure among soil samples under different vegetation reconstruction modes (Figure 3). PCoA revealed that 34.50% of the total variation in the bacterial community could be explained by the first two principal coordinates. The first and second axis explained 26.10% and 8.41% of the variance, respectively. Samples from CF sites were separated from those from the GL and BL sites along the first principal coordinate axis, and the dramatic separation of CF and BF is presented in the second axis. However, samples from the GL and BL sites were close to each other (Figure 3A). PCoA revealed that 32.30% of the total variation in the fungal community could be explained by the first two principal coordinates. The CF was separated from the GL and BL sites along the first axis and the CF sites were separated from the BF site along the second axis (Figure 3B).

**FIGURE 4:**
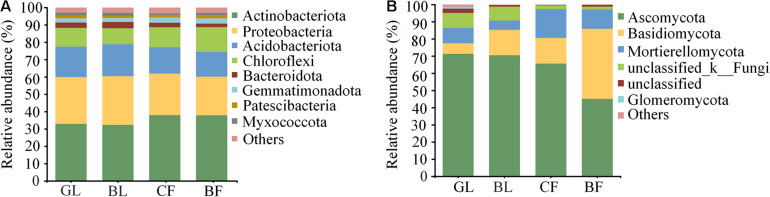
Relative abundance of soil bacterial **(A)** and fungal **(B)** communities at the phylum level.

**FIGURE 5:**
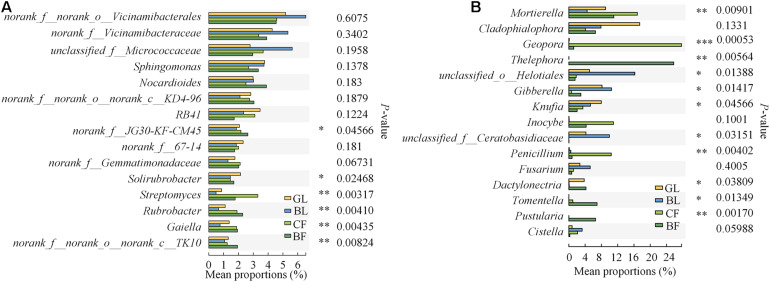
Relative abundance of the top 15 bacterial **(A)** and fungal **(B)** genera that showed significant differences among rehabilitation modes. Kruskal–Wallis *H* test was used to evaluate the significance of differences between the indicated groups. *P < 0.05, **P < 0.01, ***P < 0.001.

Our study found that different vegetation reconstruction modes significantly affected the dominant soil bacteria and fungi phyla and genus in the mining areas of the Loess Plateau. The dominant phyla of soil bacterial communities were Actinobacteriota (32.4–38.0%), Proteobacteria (22.3–28.1%), and Acidobacteriota (14.3–18.5%) under different vegetation reconstruction modes, wherein Actinobacteriota in GL and BL were significantly lower than those in CF and BF. However, the abundance of Proteobacteria in BL was 20.6% higher than that in BF (Figure 4A). Ascomycota (45.1–71.3%), Basidiomycota (6.1–40.8%), and Mortierellomycota (5.5–16.9%) were the dominant phyla for soil fungal communities. The relative abundance of Basidiomycota in BF soil is significantly lower than that in NF. The relative abundance of Ascomycetes in GL, BL, and CF was significantly higher than that in BF (*P* < 0.05), and the abundance of Basidiomycota in GL, BL, and CF was significantly lower than that in BF (Figure 4B). Members of the genera *Solirubrobacter*, *Streptomyces*, *Rubrobacter*, and *Gaiella* varied significantly under different vegetation reconstruction modes. The abundance of *Streptomyces* in GL and BL was significantly lower than that in CF and BF (*P* < 0.05, Figure 5A). Among different vegetation reconstruction modes, saprophytic fungi such as *Mortierella* and *Cladophialophora* exhibited high relative abundance. *Gibberella* predominated the BL, and *Geopora* and *Penicillium* were dominant in CF. The relative abundance of *Thelephora* and *Tomentella* was relatively higher in BF (Figure 5B).

### Comparison Between Bacterial and Fungal Community Function Among Vegetation Reconstruction Modes

**FIGURE 6:**
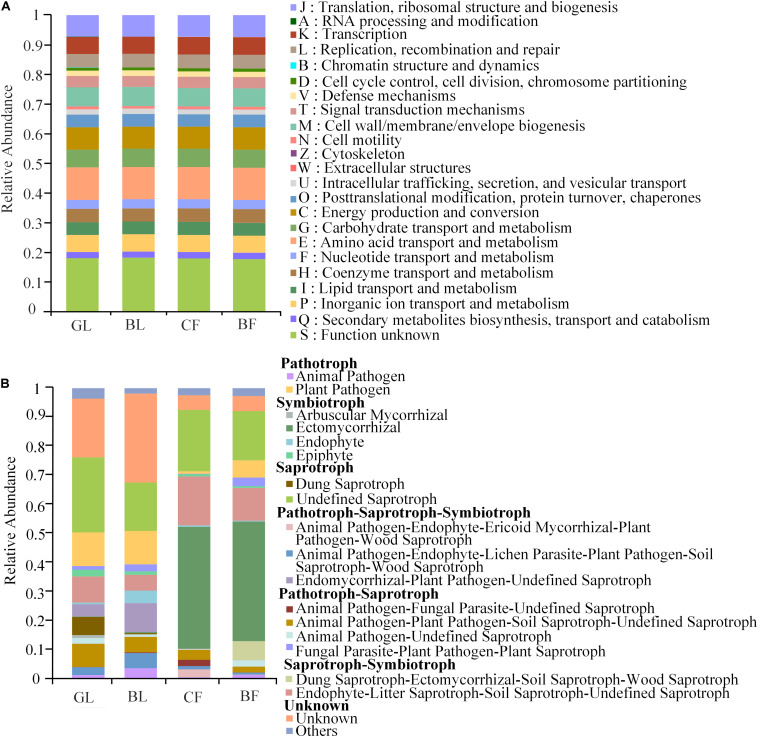
Variations in the composition of bacterial **(A)** and fungal **(B)** functional groups inferred by the PICRUSt2 and FUNGuild, respectively.

We used the PICRUSt and FUNGuild tools for a better understanding of the important roles of bacteria and fungi, respectively, in the reclaimed mining area (Figure 6A). Clusters of Orthologous Groups (COG) functional prediction analysis showed similar functional features of bacteria in different vegetation reconstruction modes (Figure 6A). These functional features of bacteria mainly included those related to energy production and conversion processes; amino acid transportation and metabolic processes; carbohydrate transportation and metabolic processes; transcription processes; cell wall, membrane, and envelope biogenesis; and signal transduction mechanism (Figure 6A). However, we observed significant differences in functions of fungi among the different vegetation reconstruction modes (Figure 6B). Ectomycorrhizal (EcM) fungi of symbiotrophy mainly existed (40%) in CF and BF. Undefined saprotrophs existed (17.0–26.0%) in different vegetation reconstruction modes, while dung saprotroph predominated (6.0%) in GL. Besides, plant pathological conditions associated with nutrition were relatively higher (11.0%) in GL and BL.

### The Relationship Between Soil Microbial Community and Environmental Factors

**FIGURE 7:**
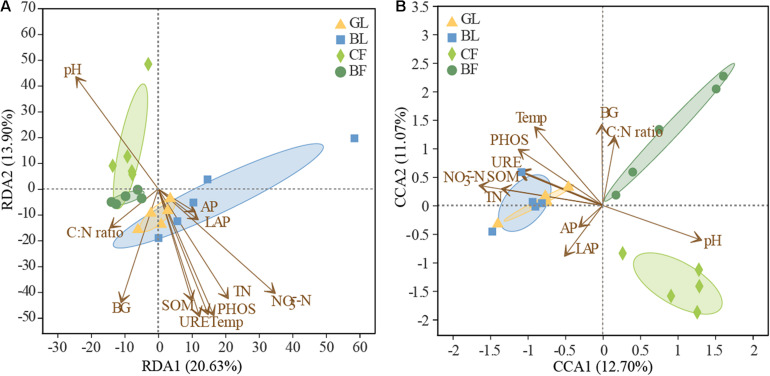
Redundancy analysis (RDA) or canonical correspondence analysis (CCA) of the relationship between soil bacterial **(A)** and fungal **(B)** community structure and edaphic factors under different vegetation patterns. Temp, soil temperature; SOM, soil organic matter; TN, total nitrogen; NO_3_^–^-N, nitrate-nitrogen; AP, available phosphorus; BG, β-1,4-glucosidase; URE, urease; LAP, leucine aminopeptidase; PHOS, phosphatase.

**FIGURE 8:**
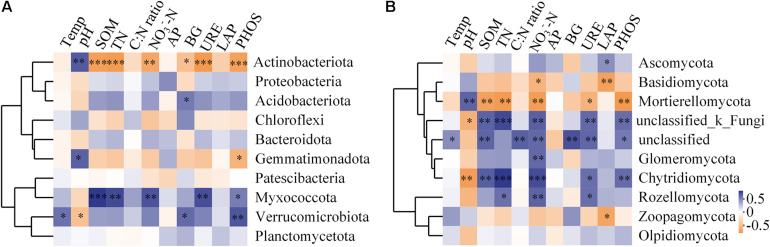
Spearman correlation heatmap of the top 10 soil bacterial **(A)** and fungal **(B)** phyla and soil properties. *X*- and *Y*-axis represent environmental factors and different phyla, respectively. Different colors of R in the extreme right show the color range of different *r* values. Significant results are indicated by *P < 0.05, **P < 0.01, ***P < 0.001.

The richness of the fungal community was significantly positively correlated with SOM, TN, NO_3_^–^-N, and urease activity (*P* < 0.05, Supplementary Table 3). The influence of soil factors under different vegetation reconstruction modes on microbial community structure was evaluated by RDA analysis (Figure 7). The soil properties could account for 34.50% of bacterial variations. Axis 1 of the RDA plot explained roughly 20.63% of the variation, while axis 2 explained a further 13.90% (Figure 7A), while soil factors could explain 23.8% of the total variation in fungal community structure (Figure 7B). In CF, bacterial and fungal community structures were found significantly positively correlated with soil pH. In GL and BL, they were significantly positively correlated with soil temperature, SOM, TN, NO_3_^–^-N, and activities of phosphatase and urease (Figures 7A,B). Fungal community structure in BF was significantly positively correlated with C to N ratio in soil and the activity of β-glucosidase (*P* < 0.05, Figure 7B). The correlation heatmap illustrated the relationship between bacterial and fungal community composition and environmental factors (Figure 8). Actinobacteriota was found to be significantly negatively correlated with SOM, TN, NO_3_^–^-N, and the activities of β-glucosidase, urease, and phosphatase and was significantly positively correlated with soil pH (*P* < 0.05, Figure 8A). Likewise, Ascomycota was significantly positively correlated with LAP activity and Basidiomycota was significantly negatively correlated with LAP activity and NO_3_^–^-N (*P* < 0.05, Figure 8B).

## Discussion

### Soil Microbial Diversity and Community Compositions

Soil microbes affect soil nutrient cycling and regulation, and they can be used as indicators of soil functions because they participate in the decomposition of soil organic matter and mineralization processes (Romaniuk et al., 2011). The change in vegetation areas under different land utilization methods might change microbial community structure and function of topsoil (Bahram et al., 2020). In this study, the dominant phyla of bacteria mainly included Actinobacteriota, Proteobacteria, and Acidobacteriota, similar to that reported in earlier studies (Chu et al., 2010; Li Y. Y. et al., 2014; Jia et al., 2020). We found that vegetation reconstruction modes significantly affected the dominant soil bacteria. Compared with those of the grassland and the brushland, the relative abundance of Actinobacteriota in coniferous forest and broadleaf forest was significantly higher, which related closely with the lower soil nutrition content. Actinobacteriota can exist in soil with low organic carbon (Sul et al., 2013) and low available nitrogen (Liu et al., 2020). The biologically available carbon is highly unstable and contains a high amount of recalcitrant lignocellulose in brushland soil (Weintraub and Schimel, 2003). Copiotrophic Proteobacteria (Kurm et al., 2017), which can utilize the highly unstable carbon in the soil (Eilers et al., 2010), was significantly higher in the brushland than in the broadleaf forest, and thus, exhibited the strategy of “r-selection,” which facilitated its growth in soil with high carbon mineralization rate (Fierer et al., 2007; Roller and Schmidt, 2015). In this study, the abundance of *Streptomyces* in grassland and brushland was significantly lower than that in coniferous forest and broadleaf forest. There are several microorganisms such as *Streptomyces* sp. strain M7 and *Chlorella vulgaris* that have been discovered by researchers that are capable of degrading the organochlorine insecticide lindane (Boudh et al., 2017). Therefore, it is suggested that *Streptomyces* can be inoculated in grassland and brushland soils and their influence on vegetation growth and physiology can be studied to enhance mine soil repair using the combination of microbes and plants.

The fungal richness of the grassland was the highest in the vegetation rehabilitation modes, which was mainly related to the high content of soil nutrients such as the SOM, TN, and NO_3_^–^-N, consistent with those reported earlier (Zeng et al., 2019). Dominant fungal phyla included Ascomycota, Basidiomycota, and Mortierellomycota. Similar results were reported by other researchers (Bahram et al., 2020), wherein Ascomycota and Basidiomycota were the ubiquitously dominant phyla (Tian et al., 2017; Zeng et al., 2019; Zhong et al., 2020) and the primary decomposers of litter and soil organic matter (Jia et al., 2020). The relative abundance of Ascomycota was relatively lower and that of Basidiomycota was relatively higher in the broadleaf forest soil, which was possibly due to the higher carbon to nitrogen ratio. Ascomycota prefers a high-quality of substrate (low C:N) (Thomson et al., 2015). Basidiomycota participates in the decomposition of low-quality substrates (high C:N) (Six et al., 2006; Thomson et al., 2015). *Penicillium* was found to help plants in the adsorption of phosphorus and promote plant growth (Whitelaw, 1999). Compared with the other three vegetation types, the abundance of *Penicillium* was dramatically higher in the coniferous forest, whose growth was thus promoted by *Penicillium*. Therefore, it is suggested that *Penicillium* can be inoculated in grassland and broadleaf forest soils and their influence on plant growth and physiology can be studied to enhance mine soil repair using the combination of microbes and plants.

### Soil Microbial Community Function

Almost all active heterotrophic fungi groups in soil could potentially contribute to organic carbon catabolism or nitrogen mineralization rate (Fierer et al., 2007). The fungal community was classified by using FUNGuild. A large scale of sequence library was categorized into those with biological meaning, including three trophic modes and 12 guilds (Nguyen et al., 2016). In this study, the functional characteristics of fungal communities among vegetation types varied significantly. However, the bacterial functional groups did not differ significantly. These functional features of bacteria mainly included those related to energy production and conversion processes and amino acid transportation and metabolic processes. Biotrophic fungal guilds such as plant pathogens and EcM mutualists are intimately associated with living plants, and thus, exhibited stronger and more specific plant interactions compared with most bacteria. Similar results were also reported previously (Martiny et al., 2006; Peay et al., 2013; Bahram et al., 2020), mainly because many roots of symbiotic fungi were specific to a certain tree species (Peay et al., 2008; Tedersoo et al., 2008).

Different functional characteristics of the fungal community were mainly exhibited due to the occurrence of symbiotic EcM in the forest soil, in agreement with previous studies which found that EcM ubiquitously exists in woodland soil (Buee et al., 2009; Branco et al., 2013; Urbanová et al., 2015). These four mechanisms – access to organic nutrients, accumulation of organic material and allelopathic compounds, and positive plant–soil feedback – act synergistically in EcM-dominated plant communities to maintain community monodominance over multiple generations. However, in the long run, it leads to the development of an ecosystem with low diversity and potential monopoly (Tedersoo et al., 2020). Two dominant genera of saprotrophic fungi, *Cladophialophora* and *Mortierella*, and the undefined saprotrophs were found to ubiquitously exist in the four vegetation reconstruction modes. More dung saprotrophs predominated the grassland possibly due to the important function of fungi in the decomposition process (Peay et al., 2008). Saprotrophic fungi promote the formation of soil organic matter and enhance nutrient cycling rate (Phillips et al., 2014), which has also been reported in previous fungal function prediction results (Nguyen et al., 2016). There were more plant pathotrophic pathogens in the grassland and the brushland, illustrating a high potential risk of disease. Due to the limitation of functional prediction analysis of PICRUSt2 and FUNGuild, the functions of related bacteria and fungi were only be preliminarily predicted and may be further verified by metagenomic analysis in future research for a better understanding of the microbial community structure of restored mining areas of the Loess Plateau, China.

### The Interaction Between Soil Microbial Community and Soil Factor

Soil microbial community structure and diversity can be controlled by many factors such as plant species (Berg and Smalla, 2009; Wang et al., 2013) and soil conditions (Lauber et al., 2008). In this study, soil pH was the primary driving factor of soil microbial community structure, which also verified that pH is a better prediction factor of soil microbial community, also reported by several earlier studies (Lauber et al., 2009; Rousk et al., 2010; Zhalnina et al., 2015). Soil nutrition status was regarded as the main factor which influences fungal community composition (Lauber et al., 2008). Our study found that bacterial and fungal community structures of the grassland and the brushland were significantly positively correlated with SOM, TN, and NO_3_^–^-N, consistent with other studies (Guo et al., 2020). The importance of the environmental factors on the shaping of microbial community structure was conspicuous. We found a positive correlation between Acidobacteria and soil β-glucosidase activity, which can degrade cellulose to provide nutrients to the soil (Pankratov et al., 2011). Saprotrophic fungi including Ascomycota and Basidiomycota were significantly correlated with LAP activity, which is related to the nitrogen cycle, indicating that both can influence the soil nitrogen cycle because all saprotrophic fungi can produce exoenzymes to mineralize carbon, nitrogen, and phosphorous in soil organic matter as well as litter (Read and Perez-Moreno, 2003).

In future studies, Proteobacteria, Acidobacteria, Basidiomycota, Ascomycota, and *Penicillium* may be inoculated into plants or soil for the ecological restoration of the mining areas. The investigation of soil microbial communities in different vegetation reconstruction modes provides an opportunity to further clarify the adaptability of microbes in mining areas and better understand the ability of microbial communities to colonize soil ecosystems in naturally fertile environments. We will further explore the related functional microbial community, enhance our understanding of self-recovery mechanisms, and serve the ecological restoration of the Loess Plateau and the construction of green mines.

## Conclusion

Vegetation reconstruction modes significantly influence soil organic matters, total nitrogen, nitrate-nitrogen, urease, and phosphatase activities as well as the richness of fungal community, wherein these factors were the highest in the grassland soil and the lowest in the coniferous soil. Different vegetation modes were found to significantly influence bacterial and fungi community structures. Bacterial and fungal community structures of the grassland and the brushland were similar and significantly different from those of the coniferous and the broadleaf forest. This might be closely related to soil pH as well as the nitrate-nitrogen content. There was a significant difference between functional groups of fungal communities in different vegetation reconstruction modes, wherein saprotrophic fungi predominated the grassland soil and symbiotrophic fungi predominated the coniferous and broadleaf forests. Concomitantly, there was no dramatic difference in functional groups of bacterial communities of different vegetation reconstruction modes. Our study suggests that shifts in vegetation type may strongly alter the mining soil microbiome structure and function. The PICRUSt and FUNGuild prediction analyses had certain limitations; therefore, subsequent studies combining metagenomic sequencing and analysis of functional genes need to be performed for a better understanding of the functions of the bacterial and fungal communities in the mining ecosystems.

## Data Availability Statement

The datasets presented in this study can be found in online repositories. The names of the repository/repositories and accession number(s) can be found in the article/Supplementary Material.

## Author Contributions

JZ, JM, and FC collected the samples. JZ, YY, SZ, and HY performed the experiments. JZ analyzed the data and along with FC contributed to the writing of the manuscript. All authors were involved in the study design and contributed to the writing of the manuscript.

## Conflict of Interest

The authors declare that the research was conducted in the absence of any commercial or financial relationships that could be construed as a potential conflict of interest.

## Publisher’s Note

All claims expressed in this article are solely those of the authors and do not necessarily represent those of their affiliated organizations, or those of the publisher, the editors and the reviewers. Any product that may be evaluated in this article, or claim that may be made by its manufacturer, is not guaranteed or endorsed by the publisher.

## Footnotes

1
www.majorbio.com
