# Supplementary material for: Response of Soil Microbial Community to Vegetation Reconstruction Modes in Mining Areas of the Loess Plateau, China
Source: Front Microbiol. 2021 Aug 25;12:714967. doi: 10.3389/fmicb.2021.714967 (PMC8424123; doi:10.3389/fmicb.2021.714967)
Supplement: Supplementary file 1 [file Data_Sheet_1.docx]

Supplementary Material

# Supplementary Figures and Tables

## Supplementary Figures


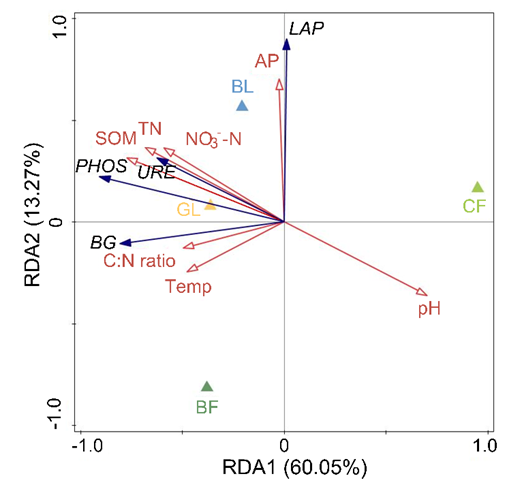


**Supplementary Figure 1.** Redundancy analysis (RDA) of the enzyme activity and physicochemical properties of soil under different vegetation types. GL, grassland; BL, brushland; CF, coniferous forest; BF, broadleaf forest. Temp, soil temperature; SOM, soil organic matter; TN, total nitrogen; NO_3_^-^-N, nitrate-nitrogen; AP, available phosphorus; BG, β-1,4-glucosidase; URE, urease; LAP, leucine aminopeptidase; PHOS, phosphatase.

## Supplementary Tables

**Supplementary Table S1** Soil physicochemical properties under different vegetation rehabilitation models.

| Parameter | Grassland (GL) | Brushland (BL) | Coniferous forest (CF) | Broadleaf forest (BF) |
| --- | --- | --- | --- | --- |
| Temperature (℃) | 27.84±0.24a | 28.92±1.47a | 24.25±0.92b | 28.33±0.20a |
| pH | 8.00±0.04b | 7.96±0.03b | 8.32±0.10a | 8.12±0.06a |
| SOM (g kg^-1^) | 19.73±2.45a | 12.11±2.28b | 5.05±0.86c | 9.09±1.27bc |
| Total N (g kg^-1^) | 0.65±0.08a | 0.47±0.07b | 0.23±0.00c | 0.29±0.03c |
| C : N ratio | 24.81±2.11a | 20.45±1.58a | 18.64±2.44a | 25.85±4.75a |
| NO_3_^-^-N(mg kg^-1^) | 8.25±0.71a | 6.41±0.66b | 1.12±0.31c | 2.10±0.23c |
| AP (mg kg^-1^) | 6.742±0.63b | 11.31±1.68a | 8.91±0.74ab | 7.37±0.44b |

^a^ SOM, soil organic matter; NO_3_^-^-N, nitrate nitrogen; AP, available phosphorus.

^b^ Values are presented as mean ± standard error (n = 5).

^c^ Different lowercase letters indicate a significant difference (*P* < 0.05) between different treatment,based on a one-way ANOVA followed by an LSD test.

**Supplementary Table S2** Bacterial and fungal alpha-diversity measures under different rehabilitation models.

| Treatment | Bacterial community | | Fungal community | |
| --- | --- | --- | --- | --- |
|  | Richness | Shannon index | Richness | Shannon index |
| Grassland (GL) | 1479.00±34.43a | 6.73±0.03a | 252.60±17.87a | 3.56±0.08a |
| Brushland (BL) | 1436.60±85.89a | 6.53±0.13a | 196.80±23.86ab | 2.98±0.40a |
| Coniferous forest (CF) | 1289.20±58.01a | 6.50±0.09a | 124.40±8.93c | 2.78±0.19a |
| Broadleaf forest (BF) | 1379.00±21.79a | 6.67±0.03a | 165.00±26.61bc | 2.78±0.29a |

^a^ Values are presented as mean ± standard error (n = 5).

^b^ Different lowercase letters indicate a significant difference (*P* < 0.05) between different treatment, based on a one-way ANOVA followed by an LSD test.

**Supplementary Table S3** Spearman’s correlation coefficients between bacterial and fungal alpha diversity indices and soil parameters.

| Soil parameters | Bacterial | | Fungal | |
| --- | --- | --- | --- | --- |
|  | Richness | Shannon index | Richness | Shannon index |
| Temperature | 0.39 | 0.23 | 0.26 | 0.03 |
| pH | -0.32 | -0.39 | -0.57 | -0.43 |
| SOM | 0.45 | 0.58 | **0.72 *** | 0.49 |
| TN | 0.59 | 0.64 | **0.68 *** | 0.57 |
| C : N ratio | 0.02 | 0.13 | 0.27 | 0.01 |
| NO_3_^-^-N | 0.61 | 0.48 | **0.67 *** | 0.51 |
| AP | 0.01 | -0.15 | -0.14 | 0.00 |
| BG | 0.34 | 0.45 | 0.57 | 0.20 |
| URE | 0.62 | 0.48 | **0.69 *** | 0.39 |
| LAP | -0.01 | -0.08 | 0.21 | 0.10 |
| PHOS | 0.47 | 0.48 | 0.66 | 0.46 |

^a^ TN, total nitrogen; NO_3_^-^-N, nitrate nitrogen; AP, available phosphorus; BG, β-1,4-glucosidase; URE, urease; LAP, leucine aminopeptidase; PHOS, phosphatase.
